# Reliability and validity of the Chinese version of the selective control assessment of the lower extremity in children with spastic cerebral palsy

**DOI:** 10.3389/fneur.2024.1458066

**Published:** 2024-09-04

**Authors:** Chunming Zhou, Yijing Chen, Wenhui Zeng, Wujie Huang, Xuefei Wu, Yating Wang, Jiamin Zhong, Jianguo Cao, Meihuan Huang

**Affiliations:** ^1^Department of Rehabilitation, Shenzhen Children's Hospital, Shenzhen, China; ^2^Rehabilitation Medicine College, Jiamusi University, Jiamusi, China

**Keywords:** cerebral palsy, spasticity, selective motor control, assessment, children

## Abstract

**Objective:**

To assess the reliability and validity of the Chinese version of the Selective Control Assessment of the Lower Extremity (SCALE) in children with spastic cerebral palsy (CP).

**Methods:**

Forty-five children with spastic CP (mean age 7.29 years, SD 2.87 years, rang 4–16 years) were recruited. Internal consistency was measured using Cronbach’s α, while test–retest and inter-rater reliability were evaluated using intra-class correlation coefficients (ICC). Construct validity was established through correlation and confirmatory factor analyses. Discriminative validity was assessed by comparing SCALE scores across varying GMFCS levels.

**Results:**

The Chinese version of SCALE demonstrates high internal consistency (Cronbach’s *α* = 0.91) and good reliability with ICCs exceeding 0.76 for test–retest and inter-rater assessments. It shows significant correlations with GMFCS (*r* = −0.76, *p* < 0.001) and Fugl-Meyer scales (*r* = 0.79, *p* < 0.001), confirming its validity. Confirmatory factor analysis supports a well-fitting model (*χ*^2^/df = 1.58, RMSEA = 0.08, SRMR <0.001, GFI = 0.98, AGFI = 0.90, CFI = 0.99, TLI = 0.98), with the latent variable’s AVE at 0.59 and CR at 0.88. Discriminative validity is evident in significant differences across GMFCS levels (*p* < 0.001), notably between levels I and II, I and III, and I and IV (*p* < 0.05).

**Conclusion:**

The Chinese version of SCALE shows good reliability and validity for assessing lower limb selective movement control in children with spastic cerebral palsy in China.

**Clinical trial registration:**

https://www.chictr.org.cn/showproj.html?proj=205380, identifier ChiCTR2400083880.

## 1 Introduction

Cerebral palsy (CP) is a common motor disorder in children caused by non-progressive brain damage during fetal or infant development (1, 2). Globally, the incidence is 2–3 per 1,000 live births (3, 4), with a prevalence of 2.46 per 1,000 live births in Chinese children aged 1–6 (1). Spastic CP, the most common subtype, includes hemiplegia, diplegia, and quadriplegia, affecting up to 87% of CP (5).

Spastic CP features loss of selective motor control (SMC), spasticity, muscle weakness and shortened muscle-tendon length (6–8). Among these, impaired SMC has the greatest impact on motor function (9). SMC allows isolated muscle activation for voluntary movements (10). Damage to the corticospinal tract and other motor pathways disrupts this control, leading to non-physiological movements and motor dysfunction (8, 11, 12).

Impaired SMC may cause slowed movements, mirror movements, abnormal coordination, and involuntary trunk movements, further restricting joint mobility and causing contractures, pain, and musculoskeletal deformities (9, 13). Accurate assessment and intervention of SMC are crucial for improving motor function in children with CP.

Primary treatments for spastic CP include motor rehabilitation and spasticity management (14, 15), which involves pharmaceutical and surgical interventions. Botulinum toxin type A is a key therapy for reducing spasticity, improving motion range and reducing discomfort (15). Surgical options like selective dorsal rhizotomy may further improve SMC (16). However, limited and conflicting evidence exists on interventions for SMC (17), and more research is needed to identify the most effective SMC interventions.

The Selective Control Assessment of the Lower Extremity (SCALE) measures lower limb voluntary control in children with spastic CP and has shown high reliability and validity (13). Despite its widespread use and translations (9, 18, 19), China lacks specialized tools for SMC assessment. This study aims to introduce and validate the Chinese version of SCALE, providing an effective assessment tool for clinicians and researchers.

## 2 Participants and methods

### 2.1 Participants

The study involved 45 patients, calculated using the formula proposed by Walter et al. (20). This approach is consistent with other validation studies, as demonstrated by Richard (21) and Kottner (22). Participants treated in both the outpatient and inpatient departments of Rehabilitation Medicine, Shenzhen Children’s Hospital, from June 2023 to December 2023 were recruited. Inclusion criteria were: diagnosis of spastic CP (spastic hemiplegia, diplegia, quadriplegia) (1); aged between 4 and 16 years; and Gross Motor Function Classification System (GMFCS) levels of I–IV. Exclusion criteria included: lower limb orthopedic surgery within the past 12 months; use of antispasmodic medication or botulinum toxin injection within the past 6 months; severe cognitive impairment hindering understanding of instructions; joint discomfort or pain; dislocated hip joint; and refusal of the participant or guardian to sign informed consent. The study was approved by the Ethics Committee of Shenzhen Children’s Hospital (Approval No. ShenErYi Ethics Approval 2,023,024), and informed consent was obtained from all participants and their guardians.

### 2.2 Translation of SCALE

The original authors of the SCALE were contacted via email and permission was obtained to translate and use the measure. SCALE was translated into Chinese using the Beaton Cross-Cultural Translation Guidelines (23). This method had been validated in other studies, such as Lavorgna et al. (24), who demonstrated the reproducibility of the Italian version of the Patient Determined Disease Steps Scale. Briefly: (1) two senior physical therapists, fluent in English and experienced in rehabilitation, independently translated the SCALE into Chinese to create a draft version. (2) Three senior physical therapy experts discussed the draft version multiple times to reach a consensus. (3) LetPub, a professional agency known for its expertise in medical translations, translated the Chinese version into English. (4) The English version was then sent to the original authors for proofreading. Based on the feedback and annotations from the original work, three senior therapists discussed and revised certain terms, resulting in the final Chinese version.

### 2.3 Methods

Lower limbs of the participants were assessed using the SCALE according to the translation assessment manual. Scores were recorded for each joint and a total score was calculated. SCALE requires children to independently perform hip, knee, ankle, subtalar, and toe movements in specific patterns. Scores are then determined based on completion and quality of the actions. Scores were recorded using a 3-point scale, and classified as normal (2 points; in which the tested joint independently completes the movement within 3 s as instructed, with a range of motion exceeding 50% of the available range and without any synergistic or mirror movements); impaired (1 point; in which the tested joint fails to complete the movement within the specified time or deviates from the movement pattern); and unable (0 points; in which the tested joint either does not move or can only move in a synergistic pattern). A higher score indicated stronger voluntary motor control capability (13).

The testing procedure involved three assessors (A, B, C) who independently evaluated the participants and simultaneously recorded the assessment process of each joint using a camera. The camera for measuring the hip joint was positioned in front of the participants, while cameras for measuring other joints were placed to the side. Each joint was recorded one by one during the test. Two weeks later, assessors re-evaluated the participants based on the video content and analyzed both results.

A senior assessor also evaluated the participants’ lower extremities according to the Fugl-Meyer Lower Extremity Assessment manual, including synergistic movement of flexors and synergistic movement of extensors in supine position, active movement with synergy and active movement without synergy in sitting position, and recorded scores as required. Scores were recorded on a 3-point scale: unable (0 points), partially completed (1 point), and fully completed (2 points) (25).

#### 2.3.1 Reliability

The reliability of the SCALE was evaluated using internal consistency test–retest reliability and inter-rater reliability. Internal consistency assessed using Cronbach’s α coefficient measured the agreement among SCALE items. Test–retest reliability compared scores from two assessments by Assessor A on the same participant at different times. Inter-rater reliability analyzed the consistency of SCALE scores among Assessors A, B, and C on the same participant.

#### 2.3.2 Validity

Criterion-related validity examined the correlation between SCALE scores, Fugl-Meyer lower extremity scores, and GMFCS levels to determine how well SCALE measures the intended constructs. The Fugl-Meyer assessment was chosen as it is a widely used clinical and research tool for evaluating motor function recovery after stroke and provides a comprehensive measure of motor impairment. The GMFCS was selected as it is a reliable and valid system to classify the severity of movement disabilities in children with cerebral palsy, making it appropriate for this study’s population. Structural validity was assessed using confirmatory factor analysis (CFA) to evaluate the rationality of the factor dimensions in the Chinese version of SCALE. The model assumed ① the scale’s factor structure consists of a single factor; ② each item belongs to the respective factor; ③ there are correlations among the items (26). Discriminative validity was assessed by comparing SCALE scores across different GMFCS levels.

### 2.4 Statistical analysis

Data were analyzed using SPSS 26.0 for statistical analysis and AMOS 21.0 for CFA. Categorical data are presented as frequencies (%), while continuous data are described as mean ± standard deviation. Inter-rater reliability and test–retest reliability of the SCALE were assessed using the intraclass correlation coefficient (ICC) based on a single measurement, absolute agreement, two-way random effects model. The interpretation of ICC coefficients was as follows: <0.50, poor reliability; 0.50–0.75, moderate reliability; 0.75–0.90, good reliability; >0.95, excellent reliability (27). Internal consistency of the SCALE was assessed using Cronbach’s α coefficient. Generally, *α* > 0.7 indicates good internal consistency, while *α* > 0.8 indicates excellent scale reliability (28).

Pearson correlation coefficients were used to analyze criterion-related validity of SCALE with Fugl-Meyer and GMFCS. CFA was performed using the maximum likelihood estimation method, and model fit was assessed using the chi-squared goodness-of-fit test (*χ*^2^), goodness-of-fit index (GFI), adjusted goodness-of-fit index (AGFI), standardized root mean square residual (RMR), comparative fit index (CFI), root mean square error of approximation (RMSEA), and Tucker–Lewis index (TLI) (29). Acceptable fit indices thresholds were GFI, AGFI, CFI, TLI ≥ 0.90, RMSEA ≤0.08, SRMR <0.08, each denoting good model fit (30, 31). The model was adjusted based on the modification index (MI) values and added residual correlations (32). Convergent validity of the SCALE was determined using the average variance extracted (AVE) and composite reliability (CR), with AVE > 0.5 and CR > 0.7 considered indicative of good convergent validity (33). The Kruskal-Wallis test was used to compare SCALE scores among different GMFCS levels, followed by *post hoc* comparisons with the Dunn test. Two-tailed tests were conducted; *p* < 0.05 was considered statistically significant.

## 3 Results

The study included 21 males and 24 females with CP, with a mean age of (7.29 ± 2.87) years. Represented CP subtypes included spastic hemiplegia (*n* = 16), spastic diplegia (*n* = 24), and spastic quadriplegia (*n* = 5). The majority of children were classified in lower levels of GMFCS, with 24 children classified as level I, 12 as level II, 5 as level III, and 4 as level IV (Table 1).

**Table 1:** Characteristics of participants.

		CP (*n* = 45)	
		*n* (%)	SCALE
Gender	Male	21 (46.7)	12.24 ± 1.08
	Female	24 (53.3)	11.88 ± 1.11
Diagnosis-distribution	Hemiplegia	16 (35.4)	15.81 ± 0.43
	Diplegia	24 (53.3)	11.33 ± 0.95
	Quadriplegia	5 (11.1)	3.40 ± 0.85
GMFCS	I	24 (53.3)	15.08 ± 0.61
	II	12 (26.7)	11.25 ± 1.30
	III	5 (11.1)	5.80 ± 1.32
	IV	4 (8.9)	4.00 ± 1.63

CP, cerebral palsy; SCALE, selective control assessment of the lower extremity; GMFCS, gross motor function classification system.

### 3.1 Reliability

The SCALE exhibited excellent internal consistency, as evidenced by a Cronbach’s α coefficient of 0.91. The test–retest reliability of the SCALE was rated as “excellent” for both individual joint scores (ICCs = 0.90–0.98) and total scores (ICCs = 0.98; Table 2). The inter-rater reliability of the SCALE was assessed among three assessors. The ICCs for individual joints ranged from 0.76 to 0.90, indicating “good reliability” in inter-rater consistency. Correspondingly, the ICCs for total scores of each lower limb ranged from 0.94 to 0.96, also demonstrating “good reliability.” Moreover, the inter-rater consistency for total scores (ICCs = 0.97) was rated as “excellent reliability” (Table 2).

**Table 2:** Intra-rater and inter-rater reliability of the Chinese version of SCALE.

	Intra-rater reliability			Inter-rater reliability		
SCALE	ICC	95%CI	*P*	ICC	95%CI	*P*
Total	0.98	0.96–0.99	*p* < 0.001	0.97	0.94–0.98	*p* < 0.001
Left hip	0.94	0.90–0.97	*p* < 0.001	0.81	0.71–0.88	*p* < 0.001
Left knee	0.97	0.94–0.98	*p* < 0.001	0.86	0.76–0.91	*p* < 0.001
Left ankle	0.96	0.93–0.98	*p* < 0.001	0.88	0.81–0.93	*p* < 0.001
Left STJ	0.94	0.88–0.94	*p* < 0.001	0.88	0.81–0.93	*p* < 0.001
Left toes	0.96	0.93–0.98	*p* < 0.001	0.9	0.84–0.94	*p* < 0.001
Left extremity	0.98	0.97–0.99	*p* < 0.001	0.96	0.93–0.96	*p* < 0.001
Right hip	0.96	0.93–0.98	*p* < 0.001	0.81	0.75–0.88	*p* < 0.001
Right knee	0.93	0.88–0.96	*p* < 0.001	0.76	0.65–0.85	*p* < 0.001
Right ankle	0.9	0.82–0.95	*p* < 0.001	0.84	0.76–0.91	*p* < 0.001
Right STJ	0.98	0.98–0.99	*p* < 0.001	0.78	0.68–0.87	*p* < 0.001
Right toes	0.98	0.97–0.99	*p* < 0.001	0.77	0.66–0.86	*p* < 0.001
Right extremity	0.98	0.95–0.99	*p* < 0.001	0.94	0.91–0.97	*p* < 0.001

SCALE, selective control assessment of the lower extremity; STJ: Subtalar joint; ICC, intraclass correlation coefficient; CI, confidence interval.

### 3.2 Validity

#### 3.2.1 Criterion-related validity

**Figure 1:**
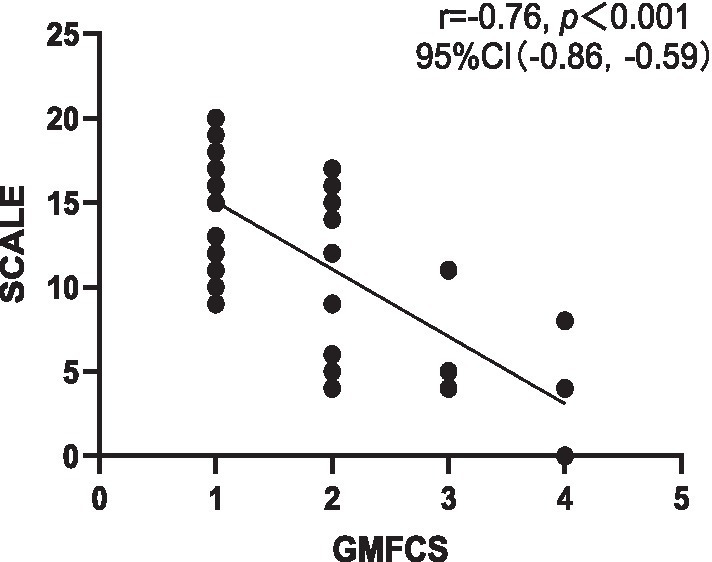
Correlation between Chinese version of SCALE and GMFCS. SCALE, selective control assessment of the lower extremity; GMFCS, gross motor function classification system; CI, confidence interval.

**Figure 2:**
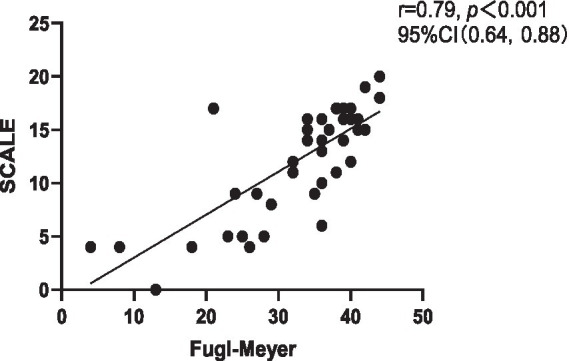
Correlation between Chinese version of SCALE and Fugl-Meyer. SCALE, selective control assessment of the lower extremity; CI, confidence interval.

Correlation analysis revealed a strong negative correlation between SCALE scores and GMFCS (*r* = −0.76, *p* < 0.001), indicating good criterion-related validity. Additionally, a strong positive correlation was observed between SCALE scores and Fugl-Meyer (*r* = 0.79, *p* < 0.001) (Figures 1, 2).

#### 3.2.2 Confirmatory factor analysis (CFA)

**Figure 3:**
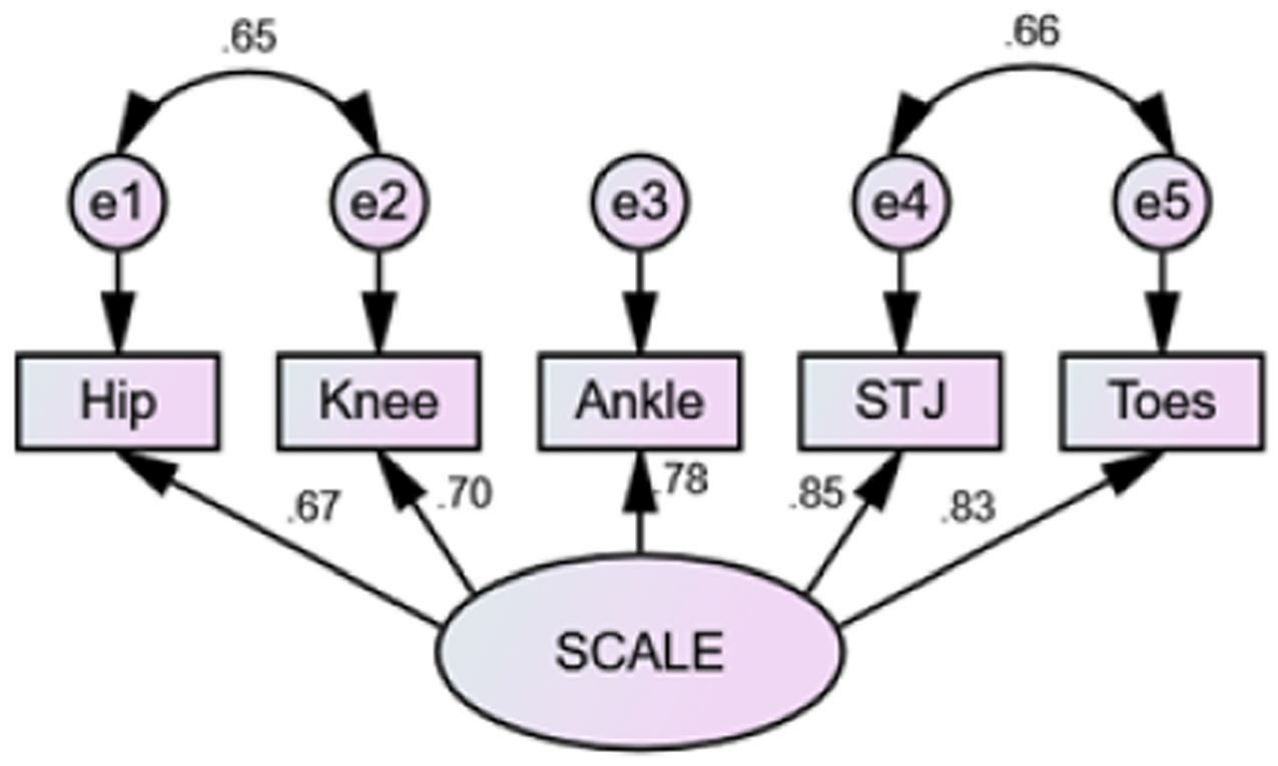
Factor model path diagram. SCALE, selective control assessment of the lower extremity; STJ, subtalar joint.

Model fit was assessed by plotting the factor model path diagram (Figure 3). The results of the original model showed *χ*^2^/df = 13.08, *p* < 0.001, RMSEA = 0.37, SRMR<0.001, and other fit indices GFI, AGFI, CFI, TLI were 0.81, 0.42, 0.37, 0.82, and 0.65, respectively, indicating poor fit. Model modification was completed sequentially based on MI values and professional knowledge. Residual correlations were added between e1 and e2, and e4, and e5 to obtain the modified model, with *χ*^2^/df = 1.58, *p* = 0.19, RMSEA = 0.08, SRMR<0.001, and other fit indices GFI, AGFI, CFI, TLI were 0.98, 0.90, 0.99, 0.98, indicating good fit. The standardized path coefficients of each item ranged from 0.67 to 0.85, all greater than 0.5. The correlations between items with freed parameters were all greater than 0.6, signifying strong correlations and supporting the theoretical assumptions of the model.

#### 3.2.3 Convergent validity

The AVE value of the latent variable was >0.5 (AVE = 0.59), and the CR value was >0.7 (CR = 0.88), both indicating good convergent validity (Table 3).

**Table 3:** Factor loading coefficients.

	Factor	Estimate	S.E	*z* (CR)	P	AVE	CR
Hip	F1	0.67					
Keen	F1	0.70	0.11	9.49	0.00		
Ankle	F1	0.78	0.25	5.98	0.00	0.59	0.88
STJ	F1	0.85	0.28	5.92	0.00		
Toes	F1	0.83	0.30	5.84	0.00		

STJ, subtalar joint; AVE, average variance extracted; CR, composite reliability.

### 3.3 Discriminant validity

There were significant differences in SCALE scores across different GMFCS levels (*H* = 22.96, *p* < 0.001). Pairwise comparisons revealed significant differences among GMFCS levels I and II (*H* = 10.94, *p* = 0.018), I and III (*H* = 22.39, *p* < 0.001), I and IV (*H* = 25.69, *p* < 0.001), but not between the others (*p* > 0.5).

## 4 Discussion

Impaired SMC is a primary factor affecting motor function in children with spastic CP (34, 35). Evaluating and treating impaired SMC in children with CP is crucial. Therefore, reliable and valid tools are of great importance for developing personalized intervention plans and evaluating treatment outcomes in clinical or research settings. Our study aimed to translate and validate the Chinese version of SCALE (SCALE-CN), to promote its use in China and provide a new method for evaluating SMC ability in children with CP.

The results of this study show that the SCALE-CN has good reliability and validity when used to evaluate children with spastic CP. The test–retest reliability (ICCs = 0.90–0.98) and inter-rater reliability (ICCs = 0.94–0.96) were both categorized as “excellent,” in line with the English, German, and Japanese versions. Thus, all exhibited satisfactory reliability (ICCs >0.90) (9, 13, 18, 19). SCALE has clear scoring rules and assessment procedures, and assessments are conducted by experienced evaluators, ensuring high test–retest reliability ICC values. Inter-rater reliability was similarly assessed by three highly experienced evaluators with extensive clinical experience. The evaluators were all from the same institution and received multiple training sessions on scoring rules and assessment procedures to ensure accurate item grasping, strict control of assessment time to avoid subject fatigue, and increased subject compliance. In this study, we used recorded videos for reliability testing. This approach helped prevent data loss and personal bias stemming from participant compliance and changes in physical health status, thereby enhancing the reliability of the data. Furthermore, this alleviated participants’ concerns about transportation and time since only one visit was required. However, video recordings using only one camera may result in uncertainties regarding certain joint movements, making it difficult for the rater to score movements accurately. To enhance the quality of video recordings, a protocol was developed to provide specific instructions for capturing each motion. Evaluators underwent training to ensure clear visibility of all motions from a video perspective.

This study satisfactorily demonstrated the criterion-related validity and structural validity of SCALE-CN. The correlation analysis demonstrated that the SCALE-CN scores had a strong correlation with GMFCS and Fugl-Meyer indicating good criterion-related validity. This accords with previous findings on the criterion-related validity of the English version (13) and other versions of SCALE (9, 18, 19). This suggests that as the severity of CP increases SMC tends to deteriorate. Furthermore, SCALE revealed significant correlations not only with GMFCS and Fugl-Meyer scores but also with muscle strength, Gross Motor Function Measure (GMFM), and gait parameters (3, 9, 36–38). In adults with CP, gross motor function is independently influenced by SMC, range of motion (ROM), and spasticity (7). This underscores the multifactorial nature of motor function in CP and the importance of addressing various aspects of motor impairment in intervention strategies.

The present study conducted confirmatory factor analysis to validate the structural validity of SCALE. Based on previous research (26) and professional knowledge, we proposed model assumptions and made successive corrections based on MI values to modify the model. The results revealed that the modified model had good fit indices consistent with the theoretical assumptions of the model, implying good structural validity and convergent validity.

To further determine the discriminant validity of SCALE-CN, we compared the scores of participants at different severity levels based on GMFCS. The overall SCALE scores showed significant differences across GMFCS levels. Specifically, there were significant score disparities between levels I and II. However, *post hoc* comparisons revealed no significant differences between others. We attribute this finding to the limited sample size. Existing studies have compared SCALE scores of children with different severity levels of spastic CP based on GMFCS (9, 18, 19) or they have compared scores between different affected limbs to assess the discriminative capability of SCALE (9). Similarly, significant differences in SCALE scores were determined in those studies.

This study had several limitations. First, all assessors came from the same institution and were familiar with the protocol; therefore, the homogeneity of the assessors may introduce institutional biases. Second, due to the predominance of children with GMFCS levels I and II in the sample and fewer children with GMFCS levels III and IV, statistical power may have been reduced while comparing SCALE scores across different GMFCS levels. Future research should further assess and characterize children with GMFCS levels III and IV to ensure data accuracy and reliability. Third, due to the cross-sectional design, we could not assess SCALE-CN’s capability to detect changes over time (e.g., responsiveness) or its suitability as an outcome measure for interventions aimed at improving SMC deficits. Future longitudinal studies are needed to investigate these aspects.

The clinical significance of these findings lies in their potential application to the rehabilitation treatment of children with CP. The demonstrated reliability and validity of the SCALE-CN support its use in clinical settings to formulate targeted intervention plans and evaluate treatment outcomes. Clinicians can use SCALE to identify specific SMC deficits and make rehabilitation strategies accordingly, potentially improving motor function and quality of life for children with CP. Furthermore, broad application of the SCALE-CN in China could enhance the standardization of SMC assessment, leading to more consistent and effective rehabilitation practices across different regions.

## 5 Conclusion

The results of this study show that the SCALE-CN is a reliable and effective tool for assessing the degree of impaired SMC in children with spastic CP. It has good reliability and validity, providing essential support for the formulation of clinical intervention plans and the evaluation of treatment outcomes. Broad application of the SCALE-CN in China could improve rehabilitation treatment and quality of life for children with CP in China.

## Data Availability

The raw data supporting the conclusions of this article will be made available by the authors, without undue reservation.
